# The severity progression of non-motor symptoms in Parkinson’s disease: a 6-year longitudinal study in Taiwanese patients

**DOI:** 10.1038/s41598-021-94255-9

**Published:** 2021-07-20

**Authors:** Yi-Chieh Chen, Rou-Shayn Chen, Yi-Hsin Weng, Ying-Zu Huang, Chiung Chu Chen, June Hung, Yi-Ying Lin

**Affiliations:** 1 Division of Movement Disorders, Department of Neurology, Chang Gung Memorial Hospital at Linkou, No. 5, Fuxing St., Guishan Dist., Taoyuan City, 333 Taiwan; 2 Medical School, Chang Gung University, Taoyuan, Taiwan; 3 Neuroscience Research Center and Department of Neurology, Chang Gung Memorial Hospital at Linkou, Taoyuan, Taiwan; 4 Healthy Aging Research Center and Medical School, Chang Gung University, Taoyuan, Taiwan

**Keywords:** Diseases of the nervous system, Neurological disorders

## Abstract

Nonmotor symptoms (NMSs) cause major burden in patients with Parkinson’s disease (PD). Previous NMSs progression studies mostly focused on the prevalence. We conducted a longitudinal study to identify the progression pattern by the severity. PD patients recruited from the outpatient clinics of a tertiary medical center were evaluated by the Unified Parkinson's Disease Rating Scale and Non-Motor Symptoms Scale (NMSS). A retrospective study with three-step analysis was performed. Step 1, the NMSs severity was compared among patients stratified by disease duration every 2 years up to 10 years. Step 2, patients with repeated tests in 2 years were categorized into 4 groups by the diseased duration of every 5 years. Step 3, the NMSS score changes in 6 years follow-up were determined, and the dosage of anti-PD drugs was compared to the NMSs severity changes. 676 patients completed the step 1 analysis, which showed a trend of NMSs worsening but not significant until the disease duration longer than 4–6 years. Furthermore, the severity did not change between repeated evaluations in 2 years in all patients. The progression became apparent after 6 years. Individual symptoms had different progression patterns and the increment of medications was independent to NMSs evolution. We demonstrated the NMSs severity progression in Taiwanese PD patients and the independence of the medications and NMSs progression.

## Introduction

An increasing amount of evidence suggests that nonmotor symptoms (NMSs) are integral to Parkinson disease (PD). These NMSs underpin the premotor stage as the initial manifestations of PD and complicate clinical management throughout the disease course. Therefore, NMSs could be a considerable determinant of quality of life. Numerous studies have used a specialised questionnaire called the Non-Motor Symptom Scale (NMSS)^1^ to measure the prevalence and severity of NMSs^2–5^. These studies have formed a general acceptance that NMSs have a high prevalence, with more than 90% of patients with PD exhibiting at least one NMS. However, little is known regarding the progression of NMSs.

Few longitudinal studies have focused on the progression of NMSs. Moreover, these studies have focused on the parameters of prevalence and frequency rather than severity. Zis et al. compared the prevalence of NMSs between drug-naïve and treated patients with PD, whose disease duration was 1.9 years or 3.7 years^6^. The 2 groups had comparable disease burden of NMSs, but their disease duration differed by 2 years. This result indirectly implies the slow evolution of NMSs. A study on progression markers in the premotor phase of PD showed that the frequency of nonmotor prodromal markers did not increase within 2 years, and that the progression of motor and nonmotor markers seemed independent^7^. Erro et al. conducted a 2-year prospective clinical study of NMSs in patients with PD in Naples, Italy^8^. The results indicated that even if the prevalence of NMSs is high in the early stage of PD, the NMSs tend to remain stable during the early phase of the disease. The researchers conducted another study with the same cohort; notably, the results revealed that NMSs significantly increased between 2nd and 4th year follow-up and that each NMS had a different progression rate^9^. Antonini et al. explored the progression of NMSs in terms of motor features and quality of life. The study highlighted that patients with PD with a higher Hoehn-Yahr stage (H&Y stage) and motor score reported more NMSs, and each NMS had a variable evolution rate^10^. These studies highlight some areas that warrant further elucidation. Evaluating the progression of NMSs in terms of NMS severity, not only in terms of prevalence, and clarifying whether the progression is linear with a steadily increasing slope or a substantial change at a specific time point are necessary.

A sex difference in NMSs in Taiwanese patients with PD was identified in a previous large-scale cross-sectional study, which revealed that male patients with PD had more severe NMSs^11^. Cross-sectional studies have reported conflicting results for NMS severity in Taiwanese patients with PD compared with those in Europe and Italy. The mean NMS severity score (NMSSI) in our previous study was 39.1 ± 34.9 in male patients and 32.8 ± 33.9 in female patients^11^; however, Martinez-Martin et al. reported that the NMSSI was 47.6 ± 40.1 in male patients and 55.1 ± 43.4 in female patients^12^, and Solla et al. reported 47.0 ± 39.2 in male patients and 66.7 ± 52.2 in female patients^13^. No sex differences in nonmotor manifestations were found in 428 Chinese patients with PD^14^. Although the documented sex differences in NMSs vary between Asian and Western studies, there is notable uncertainty. A review article highlighted the importance of unifying the evaluation scales used to address sex differences and mentioned the shortage in longitudinal studies on the progression of NMSs^15^. Whether sex differences exist in the progression of NMSs, particularly in terms of NMS severity, has not been studied for Taiwanese patients with PD.

The major antiparkinsonian drugs, namely levodopa and dopamine agonists (DAs), have been reported to affect NMSs; in particular, DAs affect sleep and depression. In patients with PD who have sleep disorders, a rotigotine patch or ropinirole is associated with sleep improvement^16,17^. However, pergolide was associated with reduced sleep quality and increased awakening during the night^18^. Pramipexole can improve depressive symptoms in patients with PD after 12 weeks of usage through its direct antidepressant effect^19^. The author highlighted that the effect is attributed to reward processing and D2/3 receptor availability. However, little is known regarding how DAs affect NMSs in terms of longitudinal evolution.

We conducted a retrospective longitudinal study to explore these topics. The aims of this 6-year follow-up study on a Taiwanese PD cohort were to delineate the progression pattern of NMSs with a focus on NMS severity, compare the sex differences in NMS evolution profiles, and determine the effect of medications on NMSs in Taiwanese patients with PD.

## Materials and methods

In this retrospective study, we analysed the clinical characteristics of patients with PD with regular follow-up at a tertiary medical centre from January 2011 to December 2017. Every patient diagnosed with idiopathic PD was asked if they consented to undergoing a detailed evaluation upon returning to outpatient clinics and, if possible, every 12 to 18 months. The patients agreed to receive evaluation and provided their personal information had signed the informed consent. We recruited patients with PD who fulfilled the diagnostic criteria of the United Kingdom Parkinson’s Disease Society Brain Bank and who had no clinical profiles to suggest atypical parkinsonism. We excluded patients with a history of major brain diseases or neuroleptic usage before PD diagnosis and patients who had ever been diagnosed as having organic brain lesions. The Institutional Review Board of Chang Gung Memorial Hospital approved the study (IRB No. 201900366B0C501).

**Figure 1:**
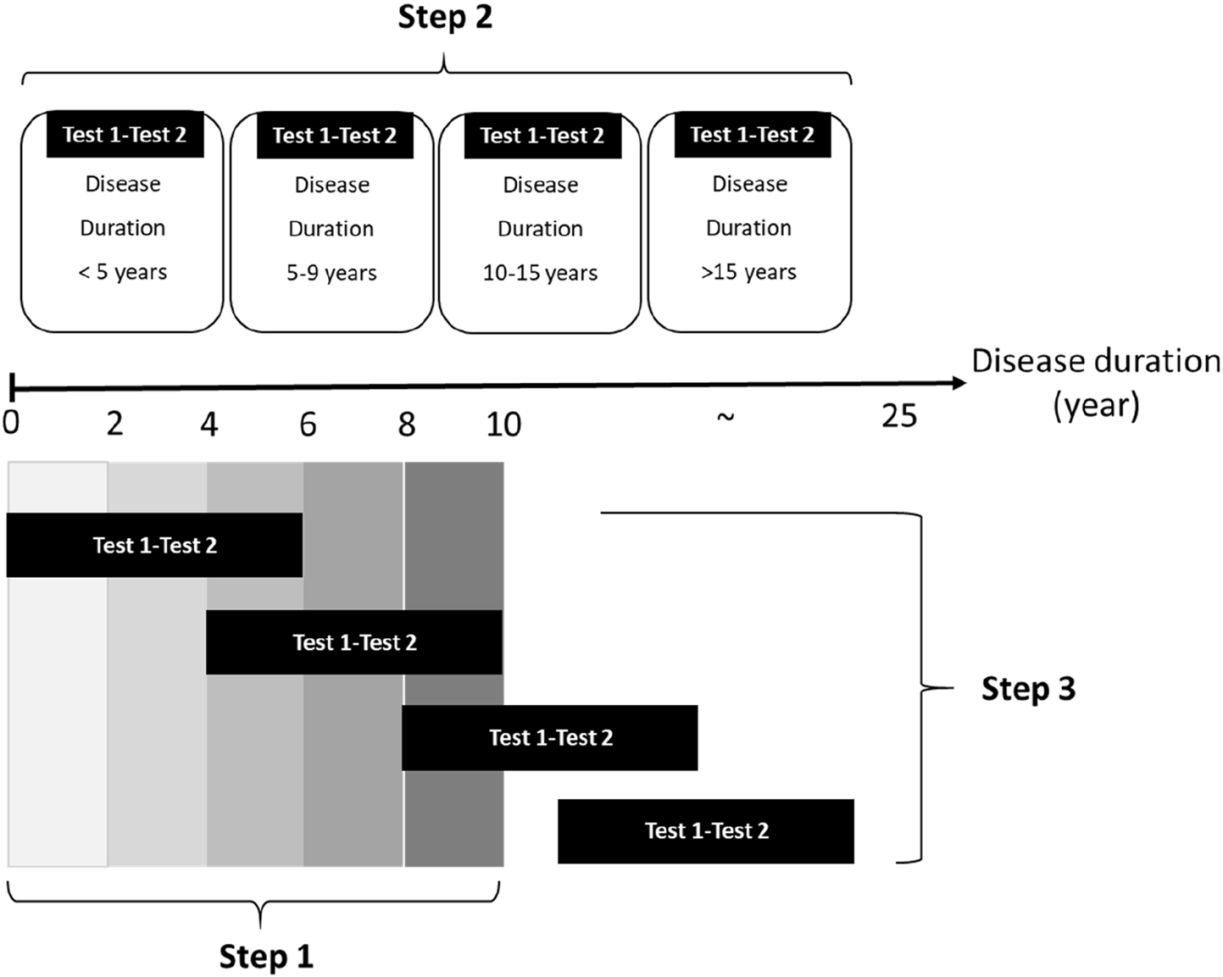
The Y axis is the disease duration, and its unit is year. We designed three steps study in our longitudinal study.

In our protocol, after diagnosed patients with idiopathic PD, we employed the NMSS for evaluating the severity of NMSs and the Unified Parkinson’s Disease Rating Scale (UPDRS) for evaluating the intensity of motor symptoms. All these methods were conducted in accordance with the approved guidelines that we followed the Declaration of Helsinki for the human rights of our participants and the Sex and Gender Equity in Research (SAGER) guidelines to discuss the different presentation in both sexes in our study. Taking into consideration the possible limitations of our patient cohort and insights from the literature, we designed several steps in our longitudinal study (Fig. 1).

In step 1, we compared NMS severity among patients stratified by disease duration of every 2 years up to 10 years. The step 1 analysis had a cross-sectional study design and involved the use of baseline data of every patient recruited into this cohort. We grouped patients by 2 years’ disease duration because some studies have reported NMS progression in 2 years, but others have not. Those with a disease duration of more than 10 years in our cohort were unevenly spread out across a wide range of disease durations of 11 to 23 years. Therefore, we set 10 years as the maximum disease duration to prevent outliers from causing unreliable statistical results.

In step 2, we selected patients who completed the evaluation twice and categorised them into 4 sets according to disease duration: less than 5 years, 5 to 9 years, 10 to 14 years, and over 15 years. By comparing the results of 2 tests from the same patient within 2 years, we could avoid bias in age, sex, or other clinical characteristics.

In step 3, we selected patients with the longest follow-up duration, which was 6 years. We interviewed those patients in person, and the number of patients was sufficient for statistical analysis. We evaluated the progression pattern in these patients and the sex effect on the progression of NMSs. UPDRS Part IV was performed to record the motor fluctuation and dyskinesia condition, while Mini-Mental State Examination (MMSE) and Montreal Cognitive Assessment (MoCA) for the cognitive function. To understand the medication effect on NMSs, we collected those patients’ antiparkinsonian drug formulas and calculated the daily levodopa dosage; levodopa equivalent dosage of DAs (LED-DA); total levodopa equivalent dosage of levodopa, DAs, and others (LED-Total); LED ratio of DAs; and LED ratio of levodopa. We used linear regression for the effect of the changing dose of medications on nonmotor symptoms in the 6 years follow up. This analysis focused on identifying parameters significantly correlated with the progression of NMSs. To further clarify the progress pattern every 2 years, we compared the changes among the 2nd, 4th, and 6th years. However, we only showed the 4th and 6th years results because of the limited patient numbers who completed all examinations for four times.

We performed one-way analysis of variance with adjusted age and sex and Bonferroni test for post hoc analysis. Linear regression was performed for the correlation of the variances. In the analysis of variance and regression model, P < 0.05 indicated statistical significance. Data from a small sample size or which did not conform to the normal distribution were analysed using the Wilcoxon signed-rank test instead of pair t test. P < 0.017 indicated a significant difference in nonparametric statistics. All statistical analyses were performed using SPSS version 24. (IBM Corp. Released 2016. IBM SPSS Statistics for Windows, Version 24.0. Armonk, NY: IBM Corp; https://www.ibm.com/tw-zh/products/spss-statistics).

## Results

A total of 820 adult patients with PD were enrolled. Among them, 343 (42.8%) were female and 477 (57.2%) were male. The mean age was 66.47 ± 9.69 years, and the mean disease duration was 5.84 ± 4.77 years. The mean score on the UPDRS Part III was 24.13 ± 0.47, the mean H&Y stage was 2.12 ± 0.03, and the mean NMSSI was 36.5 ± 34.3. The baseline demographics of all patients and those recruited in different steps are summarised in the Table 1.

**Table 1:** Demographic data of total included patients and participants in each step.

	All(n = 820)	Step 1(n = 676)	Step 2(n = 107)	Step 3(n = 72)
Disease duration (years)	5.8 ± 4.8	4.1 ± 2.4	7.5 ± 5.3	6.1 ± 3.9
Hoehn and Yahr stage	2.2 ± 0.8	2.1 ± 0.8	2.2 ± 0.7	1.8 ± 0.8
UPDRS Part III	25.4 ± 12.7	24.1 ± 12.2	23.6 ± 10.1	22.0 ± 11.8
NMSSI	36.5 ± 34.3	34.0 ± 33.3	43.1 ± 40.5	35.2 ± 32.8
**Score in each domain**				
Cardiovascular	1.8 ± 3.6	1.7 ± 3.5	2.3 ± 4.3	2.6 ± 4.2
Sleep/fatigue	7.0 ± 8.0	6.7 ± 7.9	6.2 ± 8.4	7.1 ± 8.3
Mood/apathy	6.3 ± 11.7	6.0 ± 11.6	9.1 ± 15.6	6.5 ± 12.0
Perceptual problems/Hallucination	0.8 ± 2.8	0.7 ± 2.6	1.1 ± 3.4	0.9 ± 2.2
Attention/memory	4.5 ± 6.2	4.3 ± 6.0	6.2 ± 7.5	4.0 ± 5.4
Gastrointestinal	4.7 ± 6.4	4.1 ± 5.6	4.9 ± 6.7	3.3 ± 5.2
Urinary	6.3 ± 8.4	6.0 ± 8.2	6.5 ± 9.3	5.5 ± 7.2
Sexual dysfunction	1.3 ± 3.1	1.3 ± 3.1	1.4 ± 3.2	2.4 ± 4.3
Miscellaneous	3.8 ± 5.6	3.3 ± 5.1	5.4 ± 6.3	3.5 ± 5.0

All data presented as mean ± standard deviation.

**Figure 2:**
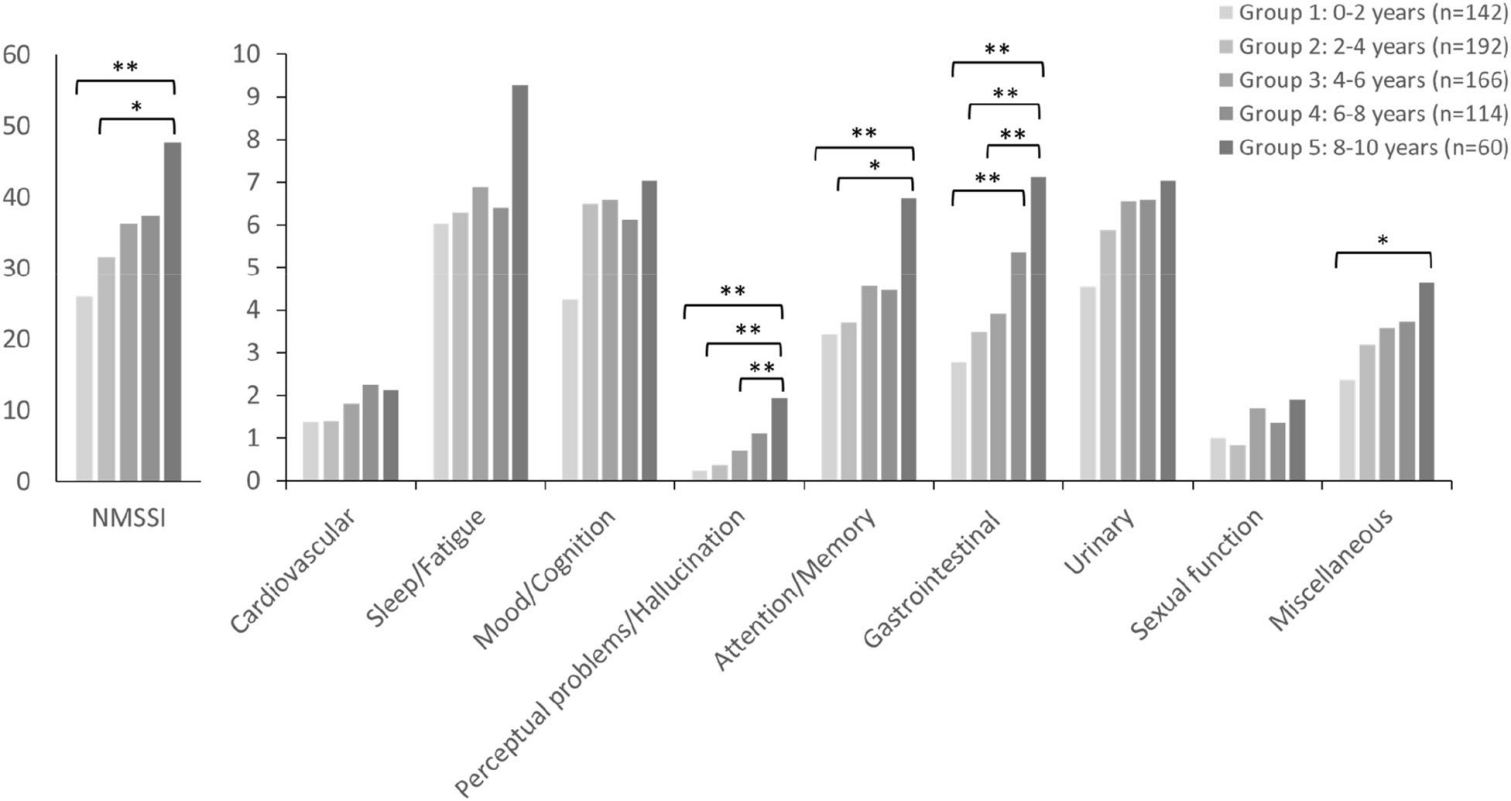
The post hoc analysis of Table 2. We only showed the significance of the comparison to group 1 to focus on the early evolution. The left figure is the total score of NMS scale and the right figure is the score of each domain. *p < 0.05; **p < 0.01.

There were 676 patients with a disease duration less than 10 years, and they were stratified into 5 groups for the step 1 analysis. The NMSSI indicated a significant increase in severity between group 1 (0–2 years) and group 3 (4–6 years), group 1 and group 4 (6–8 years), and group 1 and group 5 (8–10 years) (Table 2 and Fig. 2). These results indicated that Taiwanese patients with PD showed a trend of intensifying NMSs during the first 10 years of disease duration; however, this trend was not significant until the observation duration exceeded 4 to 6 years. Moreover, the intensification of NMSs was primarily caused by perceptual problems and hallucination (domain 4), attention and memory problems (domain 5), gastrointestinal problems (domain 6), and miscellaneous problems (domain 9). Gastrointestinal problems accounted for the greatest effect in the post hoc analysis.

**Table 2:** Comparison of NMS severity in patients with a PD disease duration of less than 10 years.

	Mean					P value
	Group 1 (0–2 years)	Group 2 (2–4 years)	Group 3 (4–6 years)	Group 4 (6–8 years)	Group 5 (8–10 years)	
Total score of NMSS	26.01	31.63	36.29	37.37	47.67	0.000**
Cardiovascular	1.37	1.38	1.8	2.24	2.12	0.192
Sleep/fatigue	6.04	6.3	6.89	6.4	9.27	0.093
Mood/cognition	4.24	6.49	6.6	6.13	7.03	0.329
Perceptual problems/hallucination	0.24	0.38	0.7	1.11	1.93	0.000**
Attention/memory	3.44	3.72	4.57	4.46	6.63	0.008**
Gastrointestinal	2.77	3.49	3.9	5.36	7.12	0.000**
Urinary	4.54	5.88	6.57	6.59	7.03	0.170
Sexual function	1.01	0.82	1.7	1.35	1.9	0.087
Miscellaneous	2.36	3.18	3.57	3.73	4.63	0.033*

Group 1: 0 to 2 years (n = 142); group 2: 2 to 4 years (n = 194); group 3: 4 to 6 years (n = 166); group 4: 6 to 8 years (n = 114); group 5: 8 to 10 years (n = 60). The mean values of each group are shown in the left 5 columns, and the *P* values calculated using one-way analysis of variance are shown in the last column. Age and sex were corrected. *P < .05; **P < .01.

A total of 107 patients were eligible for the step 2 analysis. We did not identify significant differences between the 2 tests in any of the 4 patient groups, which were grouped by disease duration in increments of 5 years. The p values were shown on Table 3 and each score change was put in the Supplementary Dataset (Table S1). This phenomenon might support the results of step 1, which indicate that the progression of NMSs could be slow in the interval of 2 years’ observation.

**Table 3:** Evolution of NMS severity in 2 years in groups of different disease duration.

Disease duration	NMSSI	Cardio vascular	Sleep/fatigue	Mood/cognition	Perceptual/hallucination	Attention/memory	Gastro-intestinal	Urinary	Sexual function	Miscellaneous
< 5 years (n = 37)	0.213	0.398	0.137	0.114	0.672	0.036	0.191	0.721	0.031	0.130
5–9 years (n = 42)	0.482	0.843	0.792	0.819	0.717	0.947	0.919	0.795	0.328	0.058
10–14 years (n = 19)	0.806	0.211	0.349	0.928	0.313	0.604	0.984	0.557	0.500	0.114
> 15 years (n = 9)	0.629	0.891	0.082	0.922	0.125	0.813	0.531	0.406	0.375	0.828

We compared the repeated test in 2 years in 107 patients. Data were obtained using the Wilcoxon signed-rank test, and *P* values are shown. *P* < .017 indicates significance.

**Figure 3:**
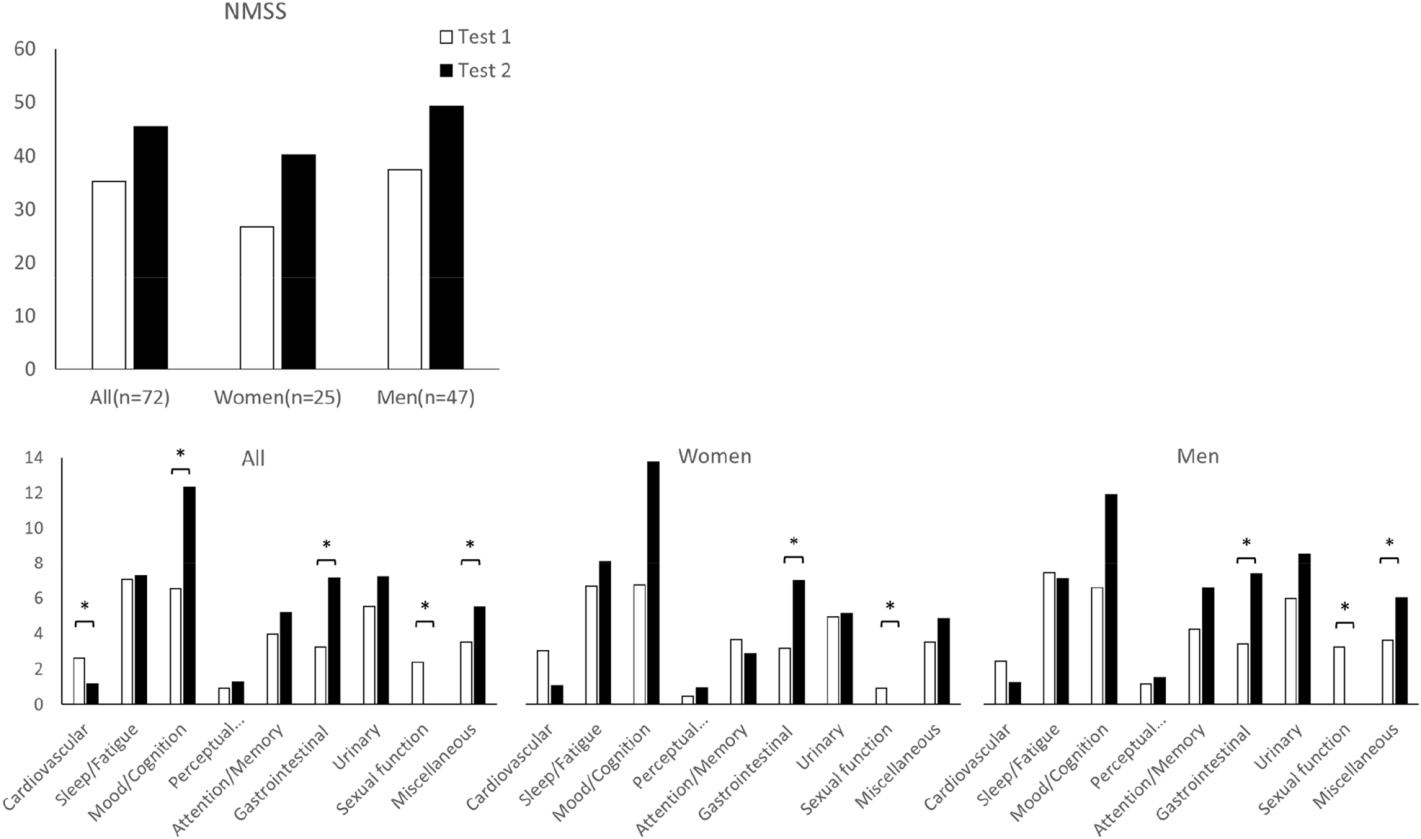
The evolution of NMSS and each domain in 6 years. Test 1 is the baseline data and Test 2 is the follow-up data. Wilcoxon signed rank test was used. P value < 0.017 indicated significance and was marked *. The upper figure showed the trend of NMSS and lower three figures showed the trend of each domain in all patients, women and men.

Step 3 was performed with 72 patients who completed a 6-year follow-up with serial evaluation. The total NMSSI exhibited a significant difference, from a score of 35.18 at baseline to 45.50 after 6-year disease progression (*P* = 0.027, Fig. 3). The NMSS domains exhibited a different trend. The mood and cognition problems (domain 3), gastrointestinal problems (domain 6), and miscellaneous problems (domain 9) demonstrated significant progression, whereas cardiovascular (domain 1) and sexual dysfunction problems (domain 8) demonstrated significant remission (Fig. 3). Among these 72 patients, 42 patients also received the 4th year examination. There were significant changes of domain 6 and domain 8 in the 4th year (both p value < 0.017), while the NMSSI showed no significance until the 6th year (p value = 0.067 in 4th year follow-up and p value = 0.014 in the 6th year follow-up). The integrated data of scores and p values were showed in Table S2 in Supplementary Datasets. To further clarify the cognitive decline, we analysed the MMSE and MoCA scores. In baseline, 14 patients had demented (MMSE score < 24) and 32 patients had MoCA scores less than 26. After 6 years, 9 patients became demented by MMSE and 12 patients’ MoCA score dropped below 26. 15.5% and 30% subjects with normal cognitive function initially became demented defined by MMSE and MoCA, respectively. In the aspect of motor complications, 9 patients suffered from dyskinesia and 21 suffered from motor fluctuation initially. After 6 years, there were 31 patients reported dyskinesia, and 24 patients reported motor fluctuation. In the regression model, NMS change was more correlated to motor fluctuation (β = 0.338, p = 0.004) rather than dyskinesia (β = 0.027, p = 0.809). Both male and female patients exhibited significant progression in domain 6 and remission in domain 8. However, male patients exhibited significant deterioration in the miscellaneous domain (domain 9), which was not noted in female patients.

Among these 72 patients, 67 had detailed records of medication prescriptions (Table 4). The UPDRS Part III score, NMSS score, LED, LED-DA, and LED-Total all showed a significant increase after the 6-years follow-up. Moreover, each NMS had a different progression direction and rate, from decreasing by 100% in the sexual dysfunction domain to increasing by 126% in the gastrointestinal symptoms domain. In our regression model, no correlation was noted between NMSSI progression and the dosage change of major antiparkinsonian medications (β = 0.086 in ΔLED-DA and β = 0.083 in Δlevodopa, both p > 0.05). We further selected patients without DA initially and were treated with DA after 6 years for further analysis (Table S3 in Supplementary Dataset). Only motor function (UPDRS Part III 19 to 27.5, p = 0.012) and sexual dysfunction (NMSS domain 8 score 2.24 to 0, p = 0.016) showed borderline significant differences. However, there were only 12 patients recruited in this portion, further study is required to confirm the result.

**Table 4:** Data of 6-year progression according to medication, motor symptoms, and NMSs.

	Test 1	Test 2	Changing ratio	P values
UPDRS PART III	22.76	31.73	39%	0.000*
LED-DA	96.6(12.7%)^	176.64(14.7%)^	83%	0.000*
Levodopa	486.12(63.8%)^	800.75(66.7%)^	65%	0.000*
LED-total	762.05	1200.47	58%	0.000*
Total score of NMSS	34.58	48.09	39%	0.008*
Cardiovascular	2.57	1.16	-55%	0.007*
Sleep/Fatigue	6.78	7.28	7%	0.837
Mood/cognition	6.51	12.25	88%	0.017*
Perceptual problems/hallucination	0.96	1.36	42%	0.306
Attention/memory	3.87	5.24	35%	0.174
Gastrointestinal	3.34	7.54	126%	0.000*
Urinary	5.58	7.6	36%	0.142
Sexual function	2.57	0	-100%	0.000*
Miscellaneous	3.75	5.66	51%	0.010*

*UPDRS* unified Parkinson’s disease rating scale; *LED* levodopa equivalent dosage; *DA* dopamine agonist.

^The percentage in parentheses is the ratio of the LED of a DA or levodopa to the total LED.

*P values < .017 indicates indicated significance by Wilcoxon signed-rank test.

## Discussion

To the best of our knowledge, this is the first long-term longitudinal study conducted in Asia that focuses on the progression of NMSs. We report 3 major findings: (1) NMS severity in Taiwanese patients with PD is initially low and progresses slowly. (2) The evolution trend of NMSs is similar for male and female patients, but some discrepancies exist. (3) The incremental dosage of DAs and levodopa had no effect on the progression of NMSs.

We identified characteristics of NMS progression in Taiwanese patients with PD. Previous studies have reported NMS progression based on the number of changes in NMS items over time; taking this into consideration, our study focused on NMS severity. The severity of NMSs in Taiwanese patients with PD is initially low and advances slowly. We have already reported in a cross-sectional study that the prevalence of NMSs in our cohort was comparable to that reported in Western studies, but the severity in our cohort was considerably lower (start low); in the present study, we further demonstrated that even when a trend of increasing NMS intensity was noted, the progression was not significant until the 6-year follow-up (go slow). Our data support the findings in Western studies on the NMS prevalence and progression, which showed that NMS tended to remain stable during the early phase of PD. Erro et al. found that NMS progression was subtle and questionable during the first 2 years of disease onset and then manifested a significant increment by the fourth year^8,9^. Our results showed the same trend of stability at the first 2-year follow-up; however, Taiwanese patients with PD presented significant deterioration at the sixth year, not at the fourth year. Our data also confirmed the concept in the literature that each NMS has a different progression rate. We identified significant increasing NMSSI in mood/ cognition domain, gastrointestinal domain, and miscellaneous domain but declines in the cardiovascular and sexual function domains. The severity of NMSs of sleep and fatigue problems, perceptual function, attention and memory deterioration, and urinary difficulty also showed an increasing tendency, but this trend did not reach statistical significance. Erro et al. reported that the percentage of patients who reported depression and difficulty concentrating became lower in the second year, whereas weight change increased, and other factors remained constant. However, in the fourth year, the prevalence of NMSs increased to 27 out of 30; among them, nausea and vomiting, sex drive, and visual hallucination were the top 3 items with increasing prevalence^8,9^. In our study, the gastrointestinal domain was not only prominent in the baseline but also progressed earlier than other domains in this longitudinal study, as the result of Italian. In contrast, our patients did not report sexual problems in the follow-up. The discrepancy in the progression or reduction items between our data and Western data may be attributable to the different lifestyles, variable diet habit, and the culture background in terms of toleration of NMSs^20,21^. Furthermore, data from China showed that NMSs in different age groups have different patterns of reduction and progression^22^. Van Wamelen et al. found that the prevalence of drooling increased only in the age group of 65 to 80 years after approximately 3 years of follow-up^23^. Explanations for this differential progression of NMSs include natural aging, intensification of motor symptoms, and the natural NMS progression.

Our study showed that male patients had a higher NMSSI but that female patients had quicker symptom progression. Picillo et al. also found that male patients presented more severe NMSs but also had higher progression velocity^24^. In terms of individual NMSS domains, our data indicated a sex difference in attention/memory, urinary, and miscellaneous domains. The sex difference in deteriorating cognition has received more attention in previous studies. Cholerton et al. suggested that male sex is a predictor of progression from no cognitive impairment to PD-MCI or PDD and reported that male patients exhibited faster cognitive decline^25^. A study conducted in Grenoble, France that reviewed patients with PD after deep brain stimulation implantation in subthalamic nucleus from 1993 to 2007 also suggested that male sex is a risk factor for developing dementia^26^. In a study on the burden on the caregivers of patients with PD, more women received antidepressant medications, and more men required cognitive enhancement treatment^27^. Even though the study did not apply the NMSS, the different medication usage implied different NMS progression trajectories in different sexes. The neurological mechanism underlying the sex difference of NMSs has not been established for at disease onset or during disease progression. However, the differences in NMS manifestation could be partially attributable to geographic and cultural influences. Further studies should distinguish the influence of each factor.

Table 4 shows each NMSS domain's changes and LED for patients whose 2 evaluations had intervals of 6 years. Our regression model suggested that the dosage of levodopa and DAs did not influence the progression of NMS severity. Although no other study has been conducted on the progression of NMS severity to offer a comparison, our results align with those of 2 longitudinal studies that focused on prevalence^9,28^. The understanding of DAs' effect on NMSs is mainly based on cross-sectional or short-term studies. The post hoc analysis of the RECOVER study showed that the rotigotine patch could ameliorate sleep problems^16^, and the International Parkinson and Movement Disorder Society suggested that pramipexole is ‘clinically useful’ in treating depression in patients with PD^29^. Motor improvement could be the confounding factor between rotigotine and sleep improvement^30^, and a double-blind study reported no significant difference in NMSs after a 12-week trial with the rotigotine patch^31^. Although the 12-week prospective study of pramipexole used a unique statistical method to exclude the possible confounding factor of motor improvement^19^, the mechanism of pramipexole’s antidepressant effect remains unclear. Whether the long-term antidepressant effect of pramipexole has the same trend is also unclear. The present study showed that the changing ratio of LED-DA reached 83% after a 6-year disease duration, but the progression of NMSSI varied in each domain. Notably, severity in the sleep/fatigue domain increased by 7%, and that in the mood/cognition domain increased by 88%. The simplest explanation for this finding is that different DAs have varying effects on different NMSs; however, a review article summarising 3 studies that compared the effect on sleep profile by switching an oral DA to a rotigotine patch indicated that there were no changes among the 3 classes of DAs^32^. In our DA-naïve group, beside motor function, only sexual domain reached borderline statistical significance. However, due to small sample size (n = 12), this data could not give solid conclusion. We speculate that even DAs have positive effect on NMSs; it could be short-term effect. The disease progression per se is the primary determinant of NMSs worsening. Well-designed, prospective, large-scale studies with long-term follow-up on how different DAs affect NMSs are crucial.

This study has some limitations. Although we initially included 820 patients with PD, only 72 patients underwent a complete 6-year follow-up. Reasons included transfer to local medical departments after diagnosis confirmation, disability to come back after motor function deterioration, and refusal to retake questionnaires even though they were still being treated in our clinics. We grouped patients according to the DA they used, but the small sample size in each group led to unreliable statistical results. Moreover, as a clinical observation study, some confounding factors were not taken into consideration. Patients with Parkin mutation were noted to have more severe depression compared with noncarriers^33^. Deng et al. conducted a 4-year longitudinal study on PD in LRRK2 carriers and noncarriers, and their results suggested different progression patterns in mutation carriers and noncarriers^34^. The different courses of NMS progression in patients with genetic PD warrants further research.

## Supplementary Information

Supplementary Information.

## References

[1] Chaudhuri KR, Martinez-Martin P (2008). Quantitation of non-motor symptoms in Parkinson's disease. Eur. J. Neurol..

[2] Chaudhuri KR (2007). The metric properties of a novel non-motor symptoms scale for Parkinson's disease: Results from an international pilot study. Mov. Disord..

[3] Martinez-Martin P (2007). Prevalence of nonmotor symptoms in Parkinson's disease in an international setting; study using nonmotor symptoms questionnaire in 545 patients. Mov. Disord..

[4] Martinez-Martin P, Rodriguez-Blazquez C, Kurtis MM, Chaudhuri KR (2011). The impact of non-motor symptoms on health-related quality of life of patients with Parkinson's disease. Mov. Disord..

[5] Barone P (2009). The PRIAMO study: A multicenter assessment of nonmotor symptoms and their impact on quality of life in Parkinson's disease. Mov. Disord..

[6] Zis P (2015). Non-motor symptoms burden in treated and untreated early Parkinson's disease patients: Argument for non-motor subtypes. Eur. J. Neurol..

[7] Liepelt-Scarfone I (2017). Progression of prodromal motor and non-motor symptoms in the premotor phase study: 2-year follow-up data. Eur. J. Neurol..

[8] Erro R (2013). Non-motor symptoms in early Parkinson's disease: A 2-year follow-up study on previously untreated patients. J. Neurol. Neurosurg. Psychiatry..

[9] Erro R (2016). The non-motor side of the honeymoon period of Parkinson's disease and its relationship with quality of life: A 4-year longitudinal study. Eur. J. Neurol..

[10] Antonini A (2012). The progression of non-motor symptoms in Parkinson's disease and their contribution to motor disability and quality of life. J. Neurol..

[11] Chen YC (2020). Nonmotor symptoms of 820 Taiwanese patients with Parkinson's disease: An exploratory-comparative study. J. Neurol..

[12] Martinez-Martin P (2012). Gender-related differences in the burden of non-motor symptoms in Parkinson's disease. J. Neurol..

[13] Solla P (2012). Gender differences in motor and non-motor symptoms among Sardinian patients with Parkinson's disease. J. Neurol. Sci..

[14] Song Y, Gu Z, An J, Chan P, G. Chinese Parkinson Study (2014). Gender differences on motor and non-motor symptoms of de novo patients with early Parkinson’s disease. Neurol. Sci..

[15] Sauerbier A, Lenka A, Aris A, Pal PK (2017). Nonmotor symptoms in Parkinson's disease: Gender and ethnic differences. Int. Rev. Neurobiol..

[16] Trenkwalder C (2011). Rotigotine effects on early morning motor function and sleep in Parkinson's disease: A double-blind, randomized, placebo-controlled study (RECOVER). Mov. Disord..

[17] Ray Chaudhuri K (2012). Improvements in nocturnal symptoms with ropinirole prolonged release in patients with advanced Parkinson's disease. Eur. J. Neurol..

[18] Comella CL, Morrissey M, Janko K (2005). Nocturnal activity with nighttime pergolide in Parkinson disease: A controlled study using actigraphy. Neurology.

[19] Barone P (2010). Pramipexole for the treatment of depressive symptoms in patients with Parkinson's disease: A randomised, double-blind, placebo-controlled trial. Lancet Neurol..

[20] Sauerbier A, Jenner P, Todorova A, Chaudhuri KR (2016). Non motor subtypes and Parkinson's disease. Parkinsonism Relat. Disord..

[21] Sauerbier A (2017). Non-motor symptoms assessed by non-motor symptoms questionnaire and non-motor symptoms scale in Parkinson's disease in selected Asian populations. Neuroepidemiology.

[22] Ou R (2014). Characteristics of non-motor symptoms in patients with Parkinson's disease exhibiting camptocormia. Gait Posture..

[23] van Wamelen DJ (2020). Drooling in Parkinson's disease: Prevalence and progression from the non-motor international longitudinal study. Dysphagia.

[24] Picillo M (2014). Gender differences in non-motor symptoms in early Parkinson's disease: A 2-years follow-up study on previously untreated patients. Parkinsonism Relat. Disord..

[25] Cholerton B (2018). Sex differences in progression to mild cognitive impairment and dementia in Parkinson's disease. Parkinsonism Relat. Disord..

[26] Bove F (2020). Dementia and subthalamic deep brain stimulation in Parkinson disease: A long-term overview. Neurology.

[27] Dahodwala N (2018). Sex disparities in access to caregiving in Parkinson disease. Neurology.

[28] Simuni T (2018). Baseline prevalence and longitudinal evolution of non-motor symptoms in early Parkinson's disease: the PPMI cohort. J. Neurol. Neurosurg. Psychiatry.

[29] Seppi K (2011). The movement disorder society evidence-based medicine review update: Treatments for the non-motor symptoms of Parkinson's disease. Mov. Disord..

[30] Antonini A, Calandrella D, Merello M, Koutsikos K, Pilleri M (2013). Effects of rotigotine on Parkinson's disease-related sleep disturbances. Expert Opin. Pharmacother..

[31] Antonini A (2015). Effects of rotigotine transdermal patch in patients with Parkinson's disease presenting with non-motor symptoms: Results of a double-blind, randomized, placebo-controlled trial. Eur. J. Neurol..

[32] Chung SJ (2017). Switching from an oral dopamine receptor agonist to rotigotine transdermal patch: A review of clinical data with a focus on patient perspective. Expert Rev. Neurother..

[33] Song J (2020). Non-motor symptoms in Parkinson's disease patients with parkin mutations: More depression and less executive dysfunction. J. Mol. Neurosci..

[34] Deng X (2019). Four-year longitudinal study of motor and non-motor symptoms in LRRK2-related Parkinson's disease. Front. Neurol..

