# Supplementary material for: The severity progression of non-motor symptoms in Parkinson’s disease: a 6-year longitudinal study in Taiwanese patients
Source: Sci Rep. 2021 Jul 20;11:14781. doi: 10.1038/s41598-021-94255-9 (PMC8292315; doi:10.1038/s41598-021-94255-9)
Supplement: Supplementary file 1 — Supplementary Information. [file 41598_2021_94255_MOESM1_ESM.docx]

| Table S1 | | | | | | | | | | | | | | | | | | | | | | | | |
| --- | --- | --- | --- | --- | --- | --- | --- | --- | --- | --- | --- | --- | --- | --- | --- | --- | --- | --- | --- | --- | --- | --- | --- | --- |
| The scores of NMS severity in 2 years progression, grouped by different disease duration | | | | | | | | | | | | | | | | | | | | | | | | |
| Disease duration | NMSSI | | | Cardiovascular | | | Sleep/Fatigue | | | Mood/Cognition | | | Perceptual/ Hallucination | | Attention/ Memory | | Gastro-intestinal | | Urinary | | Sexual function | | Miscellaneous | |
|  | Test 1 | Test 2 | Test 1 | | Test 2 | Test 1 | | Test 2 | Test 1 | | Test 2 | Test 1 | | Test 2 | Test 1 | Test 2 | Test 1 | Test 2 | Test 1 | Test 2 | Test 1 | Test 2 | Test 1 | Test 2 |
| <5 years (n=37) | 34.05 | 27.65 | 2.08 | | 1.49 | 4.81 | | 5.49 | 5.95 | | 3.62 | 0.54 | | 0.76 | 6.14 | 3.05 | 2.62 | 4.16 | 6.19 | 6.11 | 1.65 | 0.19 | 4.08 | 2.78 |
| 5-9 years (n=42) | 39.07 | 38.81 | 1.64 | | 1.50 | 7.14 | | 7.48 | 8.17 | | 7.14 | 1.00 | | 1.05 | 5.71 | 5.29 | 4.93 | 4.52 | 6.00 | 5.88 | 0.67 | 0.38 | 3.81 | 5.57 |
| 10-14 years (n=19) | 57.16 | 50.00 | 3.53 | | 2.05 | 6.26 | | 8.05 | 11.58 | | 11.21 | 2.95 | | 1.89 | 6.74 | 5.16 | 7.00 | 6.95 | 7.89 | 6.26 | 1.84 | 1.47 | 9.37 | 6.95 |
| >15 years (n=9) | 69.78 | 73.11 | 3.89 | | 3.56 | 7.44 | | 13.11 | 21.22 | | 20.44 | 0.44 | | 2.00 | 8.11 | 7.78 | 9.11 | 10.89 | 7.44 | 5.44 | 2.56 | 1.00 | 9.56 | 8.89 |
| Note. We compared the repeated test in 2 years in 107 patients. Data were obtained using the Wilcoxon signed-rank test, and *P* values are shown. *P* < .017 indicates significance. | | | | | | | | | | | | | | | | | | | | | | | | |

| Table S2 | | | | | | |
| --- | --- | --- | --- | --- | --- | --- |
| Severity of NMS progression in 1-4-6 years | | | | | | |
|  | 1st year | 4th year | 6th year | P value (1 vs 4) | P value (4 vs. 6) | P value (1 vs.6) |
| UPDRS Part III | 21.360 | 27.450 | 31.500 | 0.000* | 0.009* | 0.000* |
| NMSSI | 32.930 | 45.310 | 45.690 | 0.067 | 0.708 | 0.014* |
| Cardiovascular | 2.380 | 1.690 | 1.380 | 0.273 | 0.989 | 0.040 |
| Sleep/Fatigue | 6.170 | 7.810 | 7.520 | 0.220 | 0.516 | 0.259 |
| Mood/Cognition | 5.710 | 8.600 | 7.900 | 0.220 | 0.767 | 0.150 |
| Perceptual/Hallucination | 1.050 | 2.400 | 1.310 | 0.119 | 0.108 | 0.264 |
| Attention/Memory | 3.980 | 5.550 | 6.690 | 0.185 | 0.096 | 0.034 |
| Gastrointestinal | 3.740 | 8.480 | 8.640 | 0.000* | 0.954 | 0.000* |
| Urinary | 5.120 | 5.520 | 6.810 | 0.665 | 0.152 | 0.170 |
| Sexual function | 2.100 | 0.190 | 0.000 | 0.001* | 1.000 | 0.000* |
| Miscellaneous | 2.690 | 5.070 | 5.430 | 0.044 | 0.537 | 0.002* |
| Note: Total patient number was 42 and the data were obtained using the Wilcoxon signed-rank test. P < .017 indicates significance and marked as *. | | | | | | |
|  |  |  |  |  |  |  |

| Table S3 | | | |
| --- | --- | --- | --- |
| Severity of NMS progression in initial DA-naïve patients | | | |
|  | Test 1(before DA) | Test 2 (After DA) | P value |
| UPDRS Part III | 19.00 | 27.50 | 0.012* |
| NMSSI | 31.33 | 35.75 | 0.915 |
| Cardiovascular | 3.17 | 1.50 | 0.219 |
| Sleep/Fatigue | 6.50 | 5.42 | 1.000 |
| Mood/Cognition | 4.83 | 5.33 | 0.867 |
| Perceptual/Hallucination | 1.08 | 0.92 | 0.875 |
| Attention/Memory | 2.33 | 3.25 | 0.327 |
| Gastrointestinal | 1.42 | 5.67 | 0.049 |
| Urinary | 4.83 | 7.50 | 0.117 |
| Sexual function | 2.42 | 0.00 | 0.016* |
| Miscellaneous | 4.75 | 6.17 | 0.992 |
| Note: Total patient number was 12 and Wilcoxon signed-rank test was used. P < .017 indicates significance and marked as *. | | | |
